# Loss of 18q Alters TGFβ Signalling Affecting Anteroposterior Neuroectodermal Fate in Human Embryonic Stem Cells

**DOI:** 10.1111/cpr.13813

**Published:** 2025-02-05

**Authors:** Yingnan Lei, Mai Chi Duong, Nuša Krivec, Charlotte Janssens, Marius Regin, Anfien Huyghebaert, Edouard Couvreu de Deckersberg, Karen Sermon, Diana Al Delbany, Claudia Spits

**Affiliations:** ^1^ Vrije Universiteit Brussel (VUB), Brussels Health Campus/Faculty of Medicine and Pharmacy, Research Group Genetics Reproduction and Development Brussels Belgium; ^2^ Department of Biochemistry Ho Chi Minh city Vietnam

## Abstract

Chromosomal abnormalities acquired during cell culture can compromise the differentiation potential of human pluripotent stem cells (hPSCs). In this work, we identified a diminished differentiation capacity to retinal progenitor cells in human embryonic stem cells (hESCs) with complex karyotypes that had in common the loss of part of chromosome 18q. Time‐course gene‐expression analysis during spontaneous differentiation and single‐cell RNA sequencing found that these variant cell lines poorly specified into anterior neuroectoderm, and, when progressing through differentiation, they yielded poorly pigmented cells, with proliferating and pluripotent cell populations. The variant cell lines showed dysregulation of TGFβ signalling during differentiation, and chemical modulation of the TGFβ pathways showed that the basis of the improper specification was due to imbalances in the anteroposterior neuroectodermal fate commitment.

## Graphical abstract

- hESCs^del18q^ exhibit delayed and impaired anterior neuroectoderm induction, leading to deficient RPE specification.
- hESCs^del18q^ lines showed dysregulation of TGFβ signalling, and endogenous overactivation of activin/nodal/TGFβ in hESCs^del18q^ leads to improper anterior/posterior neuroectodermal fate commitments.

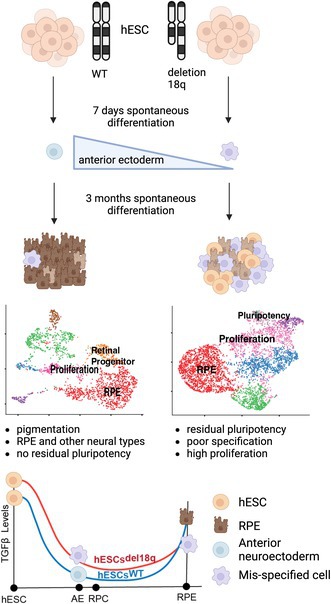

## 1 Introduction

The retinal pigment epithelium (RPE) is a monolayer of post‐mitotic, cobblestone‐shaped, pigmented and polarised cells positioned at the interface between the choriocapillaris and the sensory neural retina. The differentiation of this tissue begins shortly after gastrulation, first with the specification of the eye field from the anterior neural plate. After, the optic vesicles appear, and the RPE will emerge from their proximal part. Dysfunction, degeneration and loss of RPE cells are prominent features of retinal degenerative disease, such as Stargardt disease (SD) and age‐related macular degeneration (AMD). These conditions frequently result in significant vision loss and ultimately result in blindness [1, 2]. Phase I/II clinical transplantation trials utilising human pluripotent stem cell‐derived RPE (hPSC‐RPE) for the treatment of AMD and SD have robustly demonstrated both safety and feasibility [3, 4, 5, 6, 7].

Human pluripotent stem cells (hPSCs) are prone to acquire genomic abnormalities, which may undermine their suitability for clinical applications [8]. The most common recurrent mutations observed in hPSCs include gains of chromosomes 1q, 12p, 17, 20, X and to a lesser extent, losses of chromosomes 10p, 18q and 22p, with point mutations in *TP53* also frequently appearing [8, 9, 10, 11, 12, 13]. A critical concern regarding these genetic variants is whether and how they alter the differentiation capacity of hPSCs, thereby potentially priming differentiated cells for malignant transformation [8, 14, 15, 16, 17].

In previous work, we found that loss of 18q impairs directed neuroectodermal differentiation in human embryonic stem cells (hESCs) [18]. We worked with two cell lines carrying a derivative chromosome 18 that in both cases involves a loss of 18q, and in one instance a gain of 5q and in the other a gain of 7p. In this study, we aimed to elucidate the impact of this chromosomal abnormality on the developmental trajectory that leads to the formation of the RPE, with a special focus on the critical step of specifying the anterior and posterior plate of the neuroectodermal lineage. Further, we dissected characteristics of the derived precursors and mature RPE. To achieve this, we compared the differentiation capacity towards RPE of hESC lines with the 18q deletion (hESCs^del18q^) to genetically balanced lines (hESCs^WT^). Single‐cell RNA sequencing (scRNA‐seq) was employed to characterise cellular diversity and assess variations between hESCs^WT^ and hESCs^del18q^. Finally, treatment with specific inhibitors of key developmental pathways was used to unravel the mechanisms influencing the differentiation process.

## 2 Materials and Methods

### 2.1 hESCs Maintenance and Passaging

All lines were derived and characterised as described previously [19, 20] and are registered in the EU hPSC registry (https://hpscreg.eu/). The hESC^del18q^ were, as previously reported [18, 21], carrying derivative chromosome 18 with a loss of 18q as common aberration. VUB13^del18q^ carried a loss of q21.2qter and gain of 5q21.3qter; and VUB14^del18q^, with a loss of 18q21.32qter and gain of 7p22.3pter. Additionally, we recently found that VUB14 carry a homozygous TP53 mutation [17]. The karyotypes were assessed through shallow whole genome sequencing (Table S1 and Figure S1), at the time of cell banking. The shallow whole‐genome sequencing was carried out by the BRIGHTcore of UZ Brussels, Belgium, as previously described [22]. The cell lines were utilised within 10 passages post‐thawing to minimise the risk of (additional) genetic changes. Additionally, DNA samples were collected at the initiation of RPE differentiation to assess that the lines did not acquire the highly recurrent gains of 1q, 12p and 20q11.21 by quantitative real‐time PCR.

hESCs were maintained in NutriStem hESC XF medium (NS medium; Biological Industries) with 100 U/mL penicillin/streptomycin (P/S) (Thermo Fisher Scientific) in a 37°C incubator with 5% CO_2_, and the culture medium was changed daily. The tissue culture dishes and plates (Thermo Scientific) were coated with 10 μg/mL Biolaminin 521 (Biolamina) at 4°C and then incubated at 37°C for at least 30 min before the cells were seeded. The medium was supplemented with 10 μM Rho kinase (ROCK) inhibitor Y‐27632 (ROCKi, Tocris) for the first 24 h after passaging.

### 2.2 RPE Differentiation

The induction of RPE differentiation was initiated using a slightly modified version of the protocol from a previous publication [23, 24, 25]. hESCs were plated at 100,000 cells/cm^2^ on 20 μg/mL laminin‐521 coated dishes or plates with 10 μM ROCK inhibitor Y‐27632 during the first 24 h in NutriStem hESC XF medium. When reaching to 90% confluence, the medium was replaced with NutriStem hPSC XF GF‐free medium without basic fibroblast growth factor (bFGF) and transforming growth factor (TGFβ) with media changed every day until the timepoint for sample collection without re‐plaiting during the whole differentiation process. The pigmentation started to be visible from week 4 and pigmented areas were mechanically cut out using a sharpened glass pipette at around 90 days. The replated cells were fed twice a week with NutriStem hPSC XF GF‐free medium without bFGF and TGFβ.

### 2.3 Generation of SALL3 Knock‐Down and Overexpression Cell Lines

hESCs^WT_*SALL3*KD^ cells were generated by introducing lentiviral particles containing *SALL3*‐specific shRNAs (SigmaMISSION shRNA targeting set TRCN0000019754, TRCN0000417790) or a control shRNA plasmid into hESCs^WT^.

### 2.4 Nucleic Acids Isolation and cDNA Synthesis

RNA was isolated using RNeasy Mini and Micro kits (Qiagen) following the manufacturer's guidelines, including on‐column DNase I treatment. A minimum of 500 ng of mRNA was reverse‐transcribed into biotinylated cDNA using the First‐Strand cDNA Synthesis Kit (Cytiva) with the NotI‐d(T)18 primer. Genomic DNA was extracted with DNeasy Blood & Tissue Kit (Qiagen) following the producer's instructions. Concentration was assessed with NanoDrop 1000 (Thermo Fisher) and the samples were stored at 4°C.

### 2.5 Quantitative Real‐Time PCR (qRT‐PCR) for Gene Expression Analysis and Copy Number Assay

Quantitative real‐time PCR (qRT‐PCR) was carried out using TaqMan mRNA expression assays (Thermo Fisher Scientific) and TaqMan 2× Mastermix Plus Low ROX (Eurogentec) on a ViiA 7 thermocycler (Thermo Fisher Scientific) using the standard cycling protocol provided by the manufacturer. The relative expression of target genes was quantified using the comparative threshold cycle (Ct) method and normalised to the TaqMan *GUSB* transcript (Applied Biosystems) as the endogenous housekeeping gene. All the samples were run in triplicate, and the related TaqMan assays used in the present study are listed in Table S3. Copy‐number assays were used to control for gains of 1q, 12p and 20q11.21. Real‐time polymerase chain reaction was performed on ViiA 7 system on standard cycling conditions for copy number assays, in 2x qPCR Master Mix Low ROX (Eurogentec) with 1 μL of 20x TaqMan Copy Number Assay (Life Technologies) and 40 ng of DNA. Genomic DNA from leukocytes of healthy donor was used as a control. The copy number assays spanned the following genes: KIF14 for 1q, NANOG for 12p, ID1 for 20q11.21 and RNAseP as endogenous control (assay identifiers are listed in the Table S3). Data analysis was performed by ViiATM 7 v2.0 software or Copy Caller v2.1 (Applied Biosystems).

### 2.6 Immunostaining

Differentiated cells were first fixed in a solution of PBS containing 3.7% formaldehyde (Sigma‐Aldrich) for 15 min, permeabilized in 0.1% Triton X for 10 min (Sigma‐Aldrich) and then blocked with 10% fetal bovine serum (ThermoFisher Scientific) for 1 h at room temperature (RT). Sequentially, primary antibodies appropriately diluted in blocking solution (1:200 dilution in 10% FBS) were incubated overnight at 4°C. Thereafter, secondary antibodies conjugated to Alexa 488, Alexa 594 (1:200 dilutions in 10% FBS, Thermo Fisher Scientific) and Hoechst (12,000 dilution, ThermoFisher Scientific) were applied for 1–2 h at room temperature in the dark. Confocal images were acquired under an LSM800 (Carl Zeiss) confocal microscope at 20× magnification. The lists with antibodies can be found in Table S4.

### 2.7 Single‐Cell Suspension Preparation

Following 2 months of RPE differentiation, the RPE cells were washed at least three times with PBS to remove the dead cells and dissociated using Papain (Worthington Biochemical Corporation) for 30 mins‐1 h at 37°C following the manufacturer's guidelines. After dissociation, the cell concentration and viability was measured using Annexin V & Propidium Iodide (Thermo Fisher Scientific). Approximately 4 million cells were collected and fixed according to the manufacturer's protocol (Parse Biosciences). After fixation, the cell concentration was measured again for each suspension before being stored at −80°C. Cells were then subjected to the Single Cell Whole Transcriptome Kit v2 (Parse Biosciences) for library construction. Barcoding and sequencing library generation were performed according to the manufacturer's protocol and libraries sequenced using the high‐throughput NovaSeq (Illumina) with 20 k reads per cell.

### 2.8 Sc‐RNA Sequencing

Parse data was aligned with the ParseBiosciences‐Pipeline v0.9.6 p using the GRCh38 reference (refdata‐gex‐GRCh38‐2020‐A). Further analysis of the raw count matrices was loaded into the R package Seurat (5.3.0) for downstream analyses.

Dimension reduction was performed using RunPCA() and RunUMAP functions. The top principal components (PCs) were used to construct nearest‐neighbour graphs and identify cell clusters using the FindNeighbors() and FindClusters() functions of the Seurat R package. Single‐cell data were visualised by the “Dimplot” function. “VlnPlot” functions were used to display the RPE related gene expression levels. “DoHeatmap” functions were applied to show the heatmap. “AddModuleScore_UCell” and “DotPlot” functions were employed for the pigmentation genes expression.

### 2.9 GSEA Analysis

Highly Differentially expressed genes of each cell cluster were analysed using the Seurat “FindAllMarkers” function using “MAST” test method and genes were ranked by fold change in expression level. GSEA process analysis was performed via the clusterProfiler R package and GSEA function and the Molecular Signature Database C2 and H (MSigDB).

### 2.10 SCENIC Analysis

SCENIC analysis was carried out following the SCENIC command line protocol (Aibar et al., 2017). AUCell used the area under the curve to calculate the enrichment of the regulon across the ranking of all genes, resulting in a matrix of the activity of each regulon in each cell cluster. Downstream analyses were done in R combing the cell cluster information obtained from Seurat. Regulon specificity scores (RSS) were computed based on the cell populations clusters identified by Seurat and the AUC heatmap was plotted by the ComplexHeatmap::Heatmap function.

### 2.11 Statistical Analysis

All differentiation experiments were carried out in at least triplicate (*n* ≥ 3). All data are presented as the mean ± standard error of the mean (SEM). Statistical evaluation of differences between two groups was performed using unpaired two‐tailed *t* tests in GraphPad Prism 9 software, with *p* < 0.05 determined to indicate significance.

## 3 Results

### 3.1 hESCs^del18q^ Exhibit Impaired Anterior Neuroectoderm Induction and Poor Progression Through Poor Eye Field Induction and RPE Specification

**FIGURE 1:**
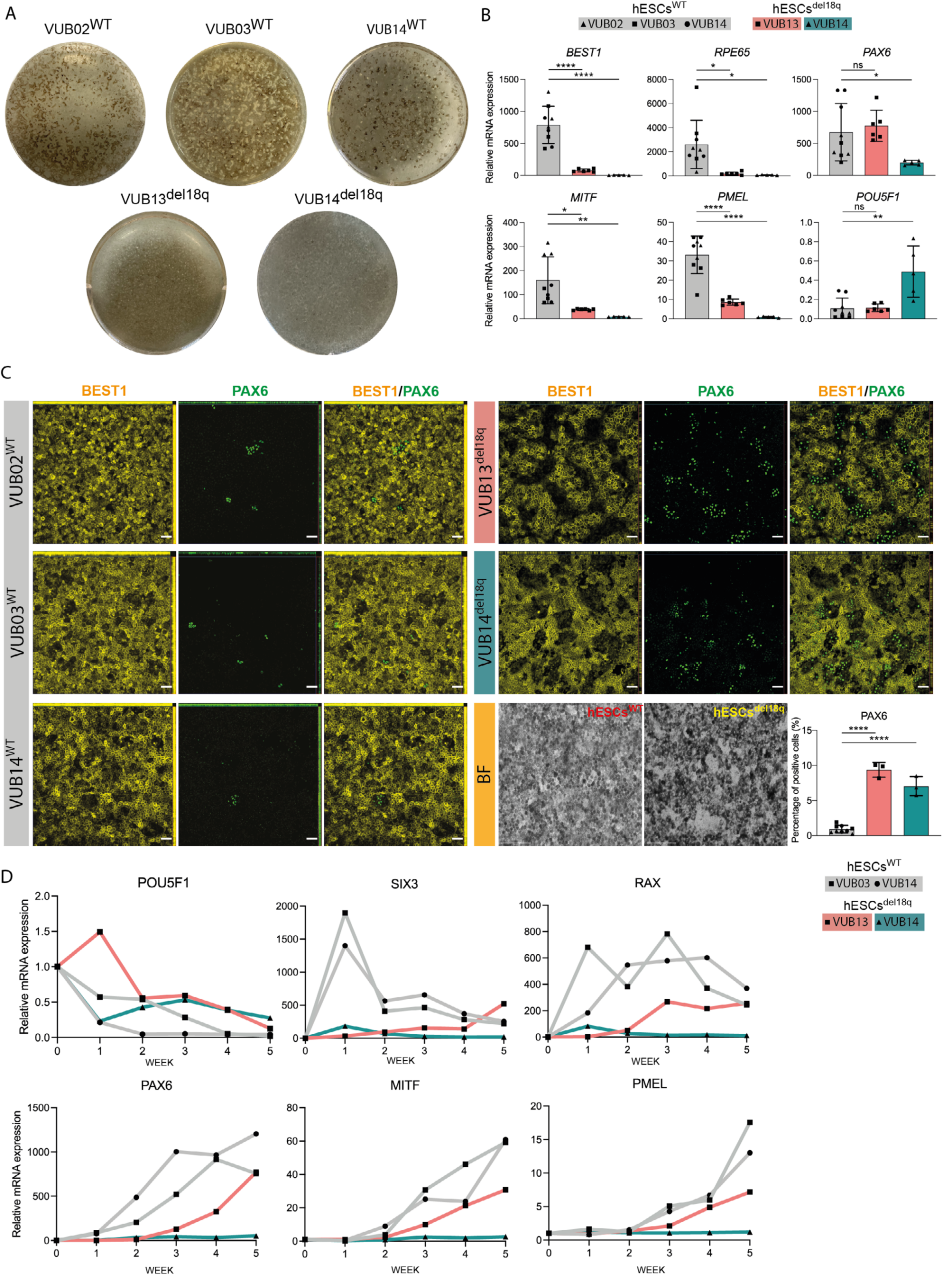
hESCs with a loss of 18q show a reduced differentiation potential to RPE and an overall poor anterior neuroectoderm induction. (A) Whole culture well images to illustrate the pigmentation observed after 3 months‐differentiation of hESCs^WT^ and hESCs^del18q^. (B) Relative mRNA expression of neuroectodermal/retinal progenitor (*PAX6*), RPE (*RPE65*, *BEST1*, *MITF*, *PMEL*) and pluripotency (*POU5F1*) markers of the dishes shown in A, prior to RPE purification. Data information: Data are shown as the means ± SEM (hESCs^WT^: *N* = 3/per line, VUB13^del18q^: *N* = 6, VUB14^del18q^: *N* = 5). (C) Staining of purified RPE for the mature RPE marker BEST1 and the neuroectodermal marker PAX6, scale bars of the 50 μm. BF (bright field) images together with counting of PAX6 positive cells (hESCs^WT^: *N* = 3/per line, VUB13^del18q^: *N* = 3, VUB14^del18q^: *N* = 3). (D) Temporal dynamics trajectory of spontaneous RPE differentiation, with relative mRNA expression of early RPE (*MITF*, *PMEL*), retinal progenitor (*PAX6, RAX* and *SIX3*) and pluripotent state (*POU5F1*) markers at 5 time points (W1, W2, W3, W4, W5). Each datapoint refers to an independent differentiation experiment and *, ** and **** represent statistical significances of *p* < 0.05, *p* < 0.01 and *p* < 0.0001 (unpaired t‐test).

To assess the effects of the 18q deletion on RPE differentiation, two hESC lines carrying a loss of chromosomes 18 (hESCs^del18q^: VUB13^del18q^ and VUB14^del18q^) and three genetically balanced lines (hESCs^WT^: VUB02^WT^, VUB03^WT^ and VUB14^WT^) were differentiated into RPE [25]. Following 3 months of RPE differentiation induction, extensive regions of the culture dishes in WT cell lines were populated with pigmented cells, a hallmark of successful differentiation to RPE. By contrast, hESCs^del18q^ exhibited only isolated and sparse patches of pigmentation, indicating a substantially diminished RPE differentiation capacity (Figure 1A). The lower differentiation efficiency was also confirmed by mRNA expression analysis of neuroectodermal (*PAX6*), RPE (*PMEL*, *MITF*, *RPE65*, *BEST1*) and pluripotency (*NANOG* and *POU5F1*) markers (Figures 1B and S2A). All the RPE markers showed a strong induction in hESCs^WT^, whereas hESCs^del18q^ exhibited significantly lower expression levels, consistent with the observed colony pigmentation patterns on the culture dishes. VUB14^del18q^ still retained expression of pluripotency markers after 3 months of differentiation (Figures 1 and S2A).

In order to further characterise the differentiated cells, the pigmented foci obtained after 90 days of differentiation were manually dissected and replated to produce a pure population of RPE cells (brightfield image in Figure 1C). Immunostaining for BEST1, ZO‐1, PMEL and PAX6 revealed that the purified RPE population obtained from hESCs^del18q^ carries a higher proportion of PAX6‐positive precursor cells and a diminished population of mature RPE cells. In hESCs^WT^‐ derived cells, we observed an average of 0.9% ± 0.4% of PAX6 positive cells, as compared to 8.1% ± 1.6% in hESCs^del18q^ (Figure 1C). The same pattern was found in the ZO‐1 and PMEL staining, which displayed negative areas in the hESCs^del18q^‐derived RPE, contrasting with the homogeneously positive staining in the hESCs^WT^‐derived RPE (Figure S2B). This suggests that the mutant lines are unable to generate a fully mature RPE.

Subsequently, we aimed at understanding if the lower degree of maturity of the cells was due to the intrinsic incapability to generate mature RPE or if there was a delay in the specification of the early eye field progenitors. We performed a time‐course experiment, collecting weekly samples from differentiating cells over a period of 5 weeks, and analysed the expression of pluripotency‐associated genes (*NANOG*, *POU5F1*), early eye field progenitor genes (*RAX*, *SIX3*), a pan‐neuroectoderm marker (*PAX6*), early RPE (*MITF*, *PMEL*) and late RPE (*BEST1*, *RPE65*) markers (Figures 1D and S2C). In hESCs^WT^, we observed a progressive downregulation of the pluripotency‐associated genes with a concomitant increase of the progenitor markers, culminating in the induction of expression of RPE markers in the final 2 weeks of the experiment. The hESCs^del18q^ lines showed a different pattern of expression. VUB13^del18q^ exhibited an overall delay in the repression of pluripotency markers, and induction of lineage‐specific genes, with lower levels of expression of RPE genes at the end point. VUB14^del18q^ never fully repressed the pluripotency genes and presented very poor induction of progenitor/RPE markers. It is worth noting that, despite the line‐specific profiles, both hESCs^del18q^ lines had significantly low induction of the anterior neuroectoderm marker *SIX3* and of the early eye field gene *RAX*. Taken together, this shows that hESCs^del18q^ do not only fail to efficiently differentiate into anterior neuroectoderm, required for eye field formation, but that if they do progress to that stage, they have a reduced capacity for RPE commitment.

### 3.2 hESCs^del18q^ Differentiate Into Cell Populations Containing Residual Undifferentiated Cells, Proliferating Cells and Immature RPEs

To gain deeper understanding on this differentiation impairment, we characterised the cells obtained after 60 days of spontaneous differentiation from hESCs^WT^ and hESCs^del18q^ by scRNA‐seq. The cell clusters were annotated based on cluster‐specific enriched markers that aligned with published cell lineage markers (listed in Table S2).

**FIGURE 2:**
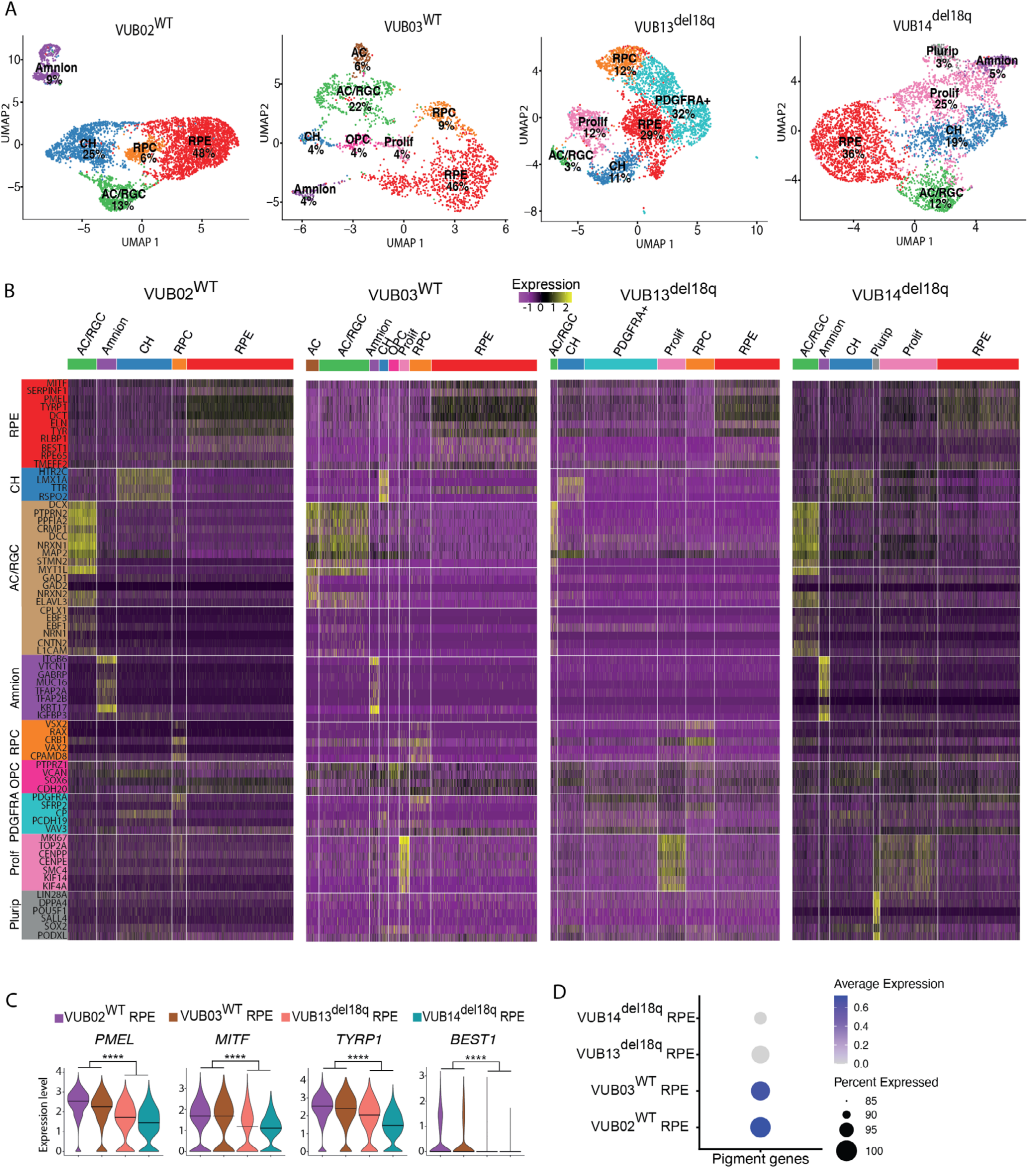
Single‐cell RNA sequencing shows that hESCs^del18q^ differentiate into cell populations containing residual undifferentiated cells, proliferating cells and immature RPEs. (A) UMAP showing the cell composition of every line after 60 days of spontaneous differentiation, with all the major lineages and their relative percentages indicated. Retinal pigment epithelium (RPE), cortical Hem (CH), amacrine Cells (AC), retinal ganglion cells (RGC), amnion cells (Amnion), retinal progenitor cells (RPC), oligodendrocyte progenitor cells (OPC), proliferating (Prolif) and pluripotent (Plurip) (B) Marker gene expression heatmap for each of the main identified cell types. (C) Violin plots showing the expression of the key RPE genes *MITF*, *PMEL*, *TYRP1* and *BEST1* in hESCs^del18q^ and hESCs^WT^ derived RPEs. Data are analysed using FindMarkers in Seurat and shown as the “avg_log2FC”. **** indicates a statistically significant difference between hESCs^WT^ and hESCs^del18q^ of *p* < 0.0001 (MAST test function from Seurat). (D) Dotplots representing the expression averages of pigmentation associated genes in the RPE subpopulations of hESCs^WT^ and hESCs^del18q^.

Clustering analysis was performed independently for each sample, revealing five populations in common between the two hESCs^WT^‐derived cells: RPE cells, retinal progenitor cells (RPC), amacrine cells or retinal ganglion cells (AC/RGC), cortical hem (CH) and amnion cells (Figure 2A). The expression of the markers for each cluster is shown in Figure 2B. The samples from the hESCs^del18q^‐derived cells overall showed similar cell populations as hESCs^WT^, but with different proportions (Figure 2A). The largest cluster of VUB13^del18q^ was marked by high expression levels of PDGFRA, SFRP2, CP, PCDH19 and VAV3. This cluster is unique to this sub‐line, and is likely composed of Müller glia cells. Notably, 48% and 46% of cells were categorised as RPE in the WT cells, compared to 29% and 36% in the two mutant lines (Figure 2A), suggesting a reduced RPE cell population in the two hESCs^del18q^. Conversely, the levels of RPE‐related genes were significantly lower in the RPE subpopulations of hESCs^del18q^ as compared to hESCs^WT^ (Figure 2C), and particularly, the pigmentation‐related genes, in line with the poor pigmentation visually observed in the hESCs^del18q^‐derived RPE (Figure 2D, pigmentation related genes listed in Table S2). hESCs^del18q^ also exhibited higher proportions of proliferating cells compared to hESCs^WT^, with proliferating cells accounting for 12% and 25% in hESCs^del18q^, versus only 4% in VUB03^WT^ and none in VUB02^WT^ (Figure 2A). Moreover, VUB14^del18q^ exhibited a cluster that remained in the pluripotent stage, in line with the retention of pluripotency‐associated gene expression found at the end point of the time‐course experiment shown in Figure 1D and the 3 months RPE induction in Figure 1B.

### 3.3 hESCs^del18q^ Derived Cells Show Deregulation of TGFβ Signalling and the Absence of Expression of RPE Master Transcriptional Regulators

**FIGURE 3:**
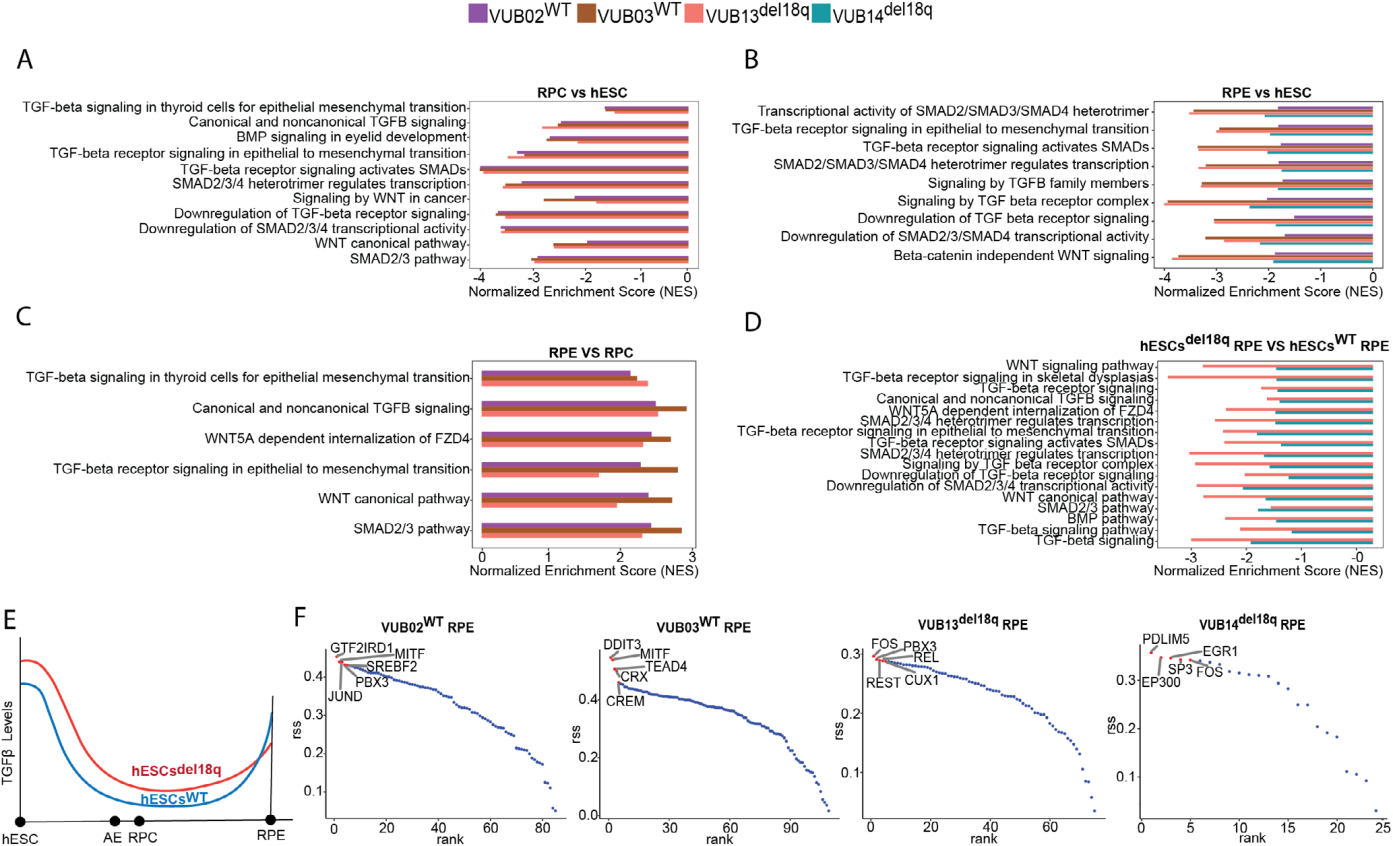
GSEA and SCENIC analysis shows deregulation of the TGFβ and Wnt signalling, and a lack of induction of RPE master regulators in cells with a loss of 18q. (A) GSEA of the RPC cell population vs. undifferentiated hESCs. (B) GSEA of the RPE cell population versus undifferentiated hESCs. (C) GSEA of the RPE versus of RPC. (D) GSEA of the RPE cell population of hESCs^del18q^ versus of hESCs^WT^. (E) Model of SMADs levels in hESCs^WT^ and hESCs^del18q^ during the whole RPE differentiation. (F) Scenic plot of the master regulators involved in the differentiation to RPE in wt and del18q cells.

We undertook differential gene expression analysis and Gene set enrichment analysis (GSEA) of the scRNA‐seq data to infer the molecular mechanisms responsible for the decreased differentiation potential of the variant lines. We first compared the pure RPC and RPE populations separately with undifferentiated hESCs (data from Couvreu et al., submitted, https://doi.org/10.21203/rs.3.rs‐5083824/v1, https://www.researchsquare.com/article/rs‐5083824/latest), and observed that both RPC and RPE, whether mutated or wild‐type, exhibited negative enrichment for gene sets associated with the TGFβ, SMAD2, SMAD3 and Wnt signalling pathways (Figure 3A,B, lists can be found in the Tables S5 and S6). Next, we compared RPE to retinal progenitor cells and found higher enrichment of these pathways in RPE (Figure 3C, lists can be found in the Table S7), indicating that these pathways are activated during the transition from progenitor cells to the RPE state. Together, these data support a specific pattern of TGFβ and Wnt pathway regulation throughout RPE differentiation; in line with a previous study on a two‐stage directed RPE differentiation model, where hESCs were initially differentiated by inhibiting TGFβ and Wnt signalling to generate anterior neural ectoderm/eye‐field cells, followed by their activation to drive further differentiation and maturation into RPE [26].

We then investigated the transcriptomic differences in the RPE populations from wt and mutant lines that could explain the reduced competence of the mutant cells to correctly and efficiently differentiate into RPE. RPE subpopulations derived from hESCs^del18q^ showed negative enrichment scores for all these TGFβ, SMAD2 and SMAD3 and Wnt signalling pathways (Figure 3D, lists can be found in the Table S8), indicating abnormally lower activation of the pathways in hESCs^del18q^ during the maturation from RPC to RPE. Figure 3E shows a schematic overview of the predicted levels of TGFβ signalling activation at each stage of differentiation in hESCs^WT^and hESCs^del18q^. Last, we employed single‐cell regulatory network inference and clustering (SCENIC) analysis to infer gene regulatory network (GRN) sets and the predict transcription factors (TFs) specifically active within each distinct RPE subpopulation. One of the most prominent GRNs in the RPE population is an MITF‐dependent network (Figure 3F). These expected regulons for MITF were active in the hESCs^WT^‐RPE, but were notably absent in the hESCs^del18q^‐RPE.

### 3.4 Endogenous Overactivation of Activin/Nodal and Wnt Signalling in hESCs^del18q^ Leads to Abnormal Responses in Anterior/Posterior Neuroectoderm Differentiation Cues

**FIGURE 4:**
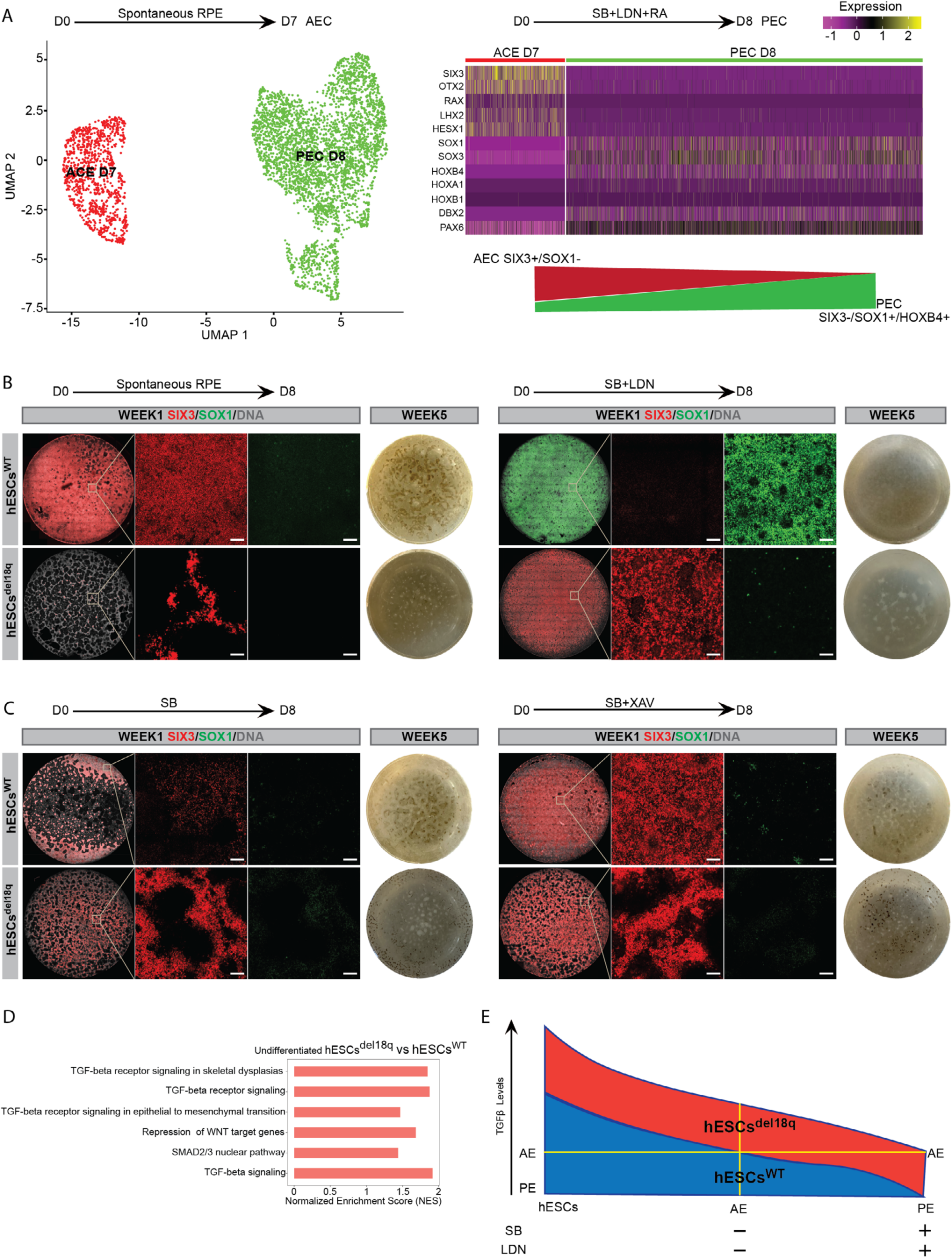
TGFβ and Wnt signalling are at the basis of an improper specification to anterior neuroectoderm and retinal progenitors in cells with a deletion of 18q. (A) (left) UMAP of the scRNA‐seq of hESCs differentiated to neuroectoderm spontaneously (ACE_D7) or through dual SMAD inhibition and retinoic acid exposure (PEC_D8) (right). The expression of posterior and anterior neuroectoderm markers in the two cell populations reveals two different cell identities. (B, C) Staining of SIX3 (red) and SOX1 (green) on week 1 of spontaneous or dual SMAD inhibition differentiation and whole 1.9 cm^2^ culture plate images to show the RPE pigmentation after 5 weeks differentiation for hESCs^WT^ and hESCs^del18q^. Scale bars of the magnification images are 100 μm (B) (left) Spontaneous and (right) dual SMAD inhibition (C) (left) SB only (Activin/Nodal inhibition) and (right) SB and XAV (Wnt inhibition). (D) Normalised enrichment scores for the GSEA for TGFβ and Wnt signalling pathways, comparing the hESCs^del18q^ to hESCs^WT^ in the undifferentiated stage. (E) Model for the proposed mechanism behind the cell fate differences. Cells in the undifferentiated state have a high activation of the TGFβ signalling. Removal of TGFβ from the culture medium is sufficient to decrease this pathway's activity to enable anterior neuroectoderm (AE) specification in genetically balanced cells. Further active inhibition of the pathway results in posterior neuroectoderm (PE). Mutant cells have higher endogenous TGFβ activation, and factor withdrawal is insufficient to activate differentiation, and additional pathway inhibition yields only the threshold for anterior neuroectoderm induction.

Finally, to unravel the mechanisms behind the differentiation impairment of hESCs^18q^, we investigated the fate of hESCs^WT^ upon spontaneous differentiation and directed neuroectoderm differentiation using Activin/Nodal and BMP4 inhibitors (dual SMAD inhibition), along with retinoic acid. We carried out scRNA‐seq at Day 7 of spontaneous differentiation and Day 8 of the directed differentiation and found that the two yield different subtypes of neuroectoderm (Figure 4A). While the cells obtained from the spontaneous differentiation induce higher levels of the anterior neuroectoderm makers *SIX3*, *OTX2*, *RAX*, *LHX2* and *HESX1*, the cells emerging from dual SMAD inhibition express rather posterior neuroectoderm genes (*SOX1*, *SOX3*, *HOXB4*, *HOXA1*, *HOXB1* and *DBX2*) (Figure 4A). *PAX6* showed to be a pan‐neuroectoderm marker, with expression in both lineages. We next sought to assess if modulation of the TGFβ and Wnt signalling (both key in the anterior/posterior neuroectoderm specification) could restore the correct specification of the hESCs^del18q^. hESCs^WT^ and hESCs^del18q^ were treated for 8 days with inhibitors of Activin/Nodal signalling (SB431542, SB), BMP4 signalling (LDN‐193189, LDN) and Wnt signalling (XAV‐939, XAV), either individually or in combination. Following 8 days treatment, a subset of cells was fixed for the assessment of the expression of the eye‐field progenitor/anterior neuroectoderm markers SIX3 and the posterior neuroectoderm marker SOX1. The remaining dishes were kept in culture for another 4 weeks and then imaged to assess for pigmentation as a proxy for successful RPE induction.

As seen from the sc‐RNAseq, in the hESCs^WT^ spontaneous differentiation without addition of factors resulted predominantly in a *SIX3*‐positive *SOX1*‐negative anterior neuroectoderm population, while hESCs^del18q^ yielded a poor overall induction to neuroectoderm, with a few *SIX3*‐positive cells and no *SOX1*‐positive cells (Figures 4 and S3A). Treatment with SB and LDN yielded posterior neuroectoderm in the WT cells, and remarkably, anterior neuroectoderm in the del18q cells. However, these anterior neuroectoderm failed to progress into pigmented RPE at week 5 (Figures 4B and S3A). Activin/nodal inhibition through SB treatment alone moderately promoted anterior neuroectoderm induction in del18q cells, which subsequently developed modest black pigmentation by week 5. In contrast, hESCs^WT^ cells exhibited a reduced population of SIX3‐positive cells following SB treatment at week 1, along with a notable decrease in pigmentation by Week 5 (Figures 4C and S3B). The addition of WNT inhibition with SB did modestly improve anterior neuroectoderm induction in del18q cells, leading to RPE pigmentation by Week 5. In comparison, hESCs^WT^ cells demonstrated efficient SIX3 induction by Week 1, but exhibited reduced efficiency in RPE induction at Week 5 (Figures 4C and S3B). Together, this supports the known key role of Activin/Nodal and Wnt signalling in the anterior/posterior axis formation, and suggests that, under normal conditions, BMP4 inhibition is necessary for the formation of posterior neuroectoderm. Conversely, del18q cells require both activin/nodal inhibition to progress to anterior neuroectoderm and Wnt inhibition to generate retinal progenitor cells able to yield correctly pigmented RPE.

Last, in previous work we carried out bulk RNA sequencing of undifferentiated hESCs and found that hESCs^del18q^ display endogenously higher activation of the TGFβ and Wnt signalling as compared to hESCs^WT^ (reanalysis shown in Figure 4D). This is in line with the GSEA analysis findings from the scRNA‐seq data in this manuscript, where TGFβ and SMAD2/3 playing an important role during the RPE development (Figure 3E). Taken together, this yields a model in which in WT cells, withdrawal of TGFβ from the culture medium is sufficient to decrease the pathway activation to induce anterior neuroectoderm. Further suppression of the pathway using BMP inhibitors (LDN) results in posterior neuroectoderm fate. In hESCs^del18q^, spontaneous differentiation does not result in sufficient suppression of the TGFβ to correctly induce any neuroectoderm specification, and the further inhibitors with LDN only suppress the pathway to meet the threshold for anterior neuroectoderm induction (Figure 4E).

The common region of 18q loss spans the gene *SALL3*, whose levels of expression have been reported to serve as a reliable indicator of hPSC differentiation bias towards either ectoderm or mesendoderm cell fates [27]. Our previous work showed that hESCs^del18q^ exhibit poor responsiveness to dual SMAD pathway inhibition for directed neuroectodermal differentiation, a deficiency that was recapitulated when *SALL3* was downregulated in wild‐type cells [18]. To explore *SALL3*'s role in regulating hESCs differentiation into the anterior and posterior neuroectoderm fate, we generated *SALL3* knockdown (KD) lines by short hairpin RNA (hESC^WT_*SALL3*KD^) alongside control lines treated with a non‐targeting short hairpin RNA (hESC^WT_NT^). After 1 week of culturing these lines under either directed neuroectoderm differentiation by dual SMAD inhibition (with SB and LDN) or in spontaneous differentiation conditions, we examined the expression of the anterior/posterior neuroectoderm markers SIX3 and SOX1 (Figure S3C). In line with our previous findings [18], *SALL3* knockdown led to diminished differentiation toward both anterior and posterior neuroectodermal fates, suggesting that *SALL3* is key in regulating overall neuroectoderm induction, but not in regulating the anterior/posterior balance.

## 4 Discussion

In this study, we present a systematic evaluation of the impact of segmental losses of chromosome 18 on the developmental trajectory of hESCs differentiating in RPE, using two hESCs^del18q^ lines along with multiple genetically balanced counterparts. Our findings unveil that hESCs harbouring an 18q deletion exhibited abnormal specification of the anterior and posterior neuroectoderm due to endogenous hyperactivation of the activin/nodal signalling pathway. This translated into a diminished capacity for RPE differentiation, manifested by sparse pigmented foci and attenuated expression of RPE‐specific markers at the endpoint of differentiation. Intriguingly, even in instances where pigmented RPE was formed, these cells exhibited poor RPE characteristics, further highlighting the profound impact of the 18q deletion on both the efficiency and quality of differentiation.

As for the mechanism responsible for this impaired differentiation, we found two key bottlenecks. We observe both an early impairment in the lineage specification of the anterior neuroectoderm, as indicated by the delayed expression of *SIX3*, and an incapacity to fully mature the retinal precursor cells to RPE. This is supported by the results of tracking the developmental process through time‐course experiments, the presence of a high proportion of proliferating and pluripotent cells in the scRNA‐seq, as well as by the significant differences in the transcriptome of the cells. Building upon these insights, we further demonstrated that modulating TGFβ and Wnt pathways through strategic inhibitor treatments could at least partially restore the RPE differentiation of hESCs^del18q^. We found that hESCs^del18q^ respond differently to the TGFβ inhibition than hESCs^WT^. In wild type lines, we found that the balance of specification of anterior and posterior neurectoderm depended on the intensity of the inhibition of the TGFβ signalling. For wild type lines, withdrawal of TGFβ from the culture medium is sufficient to induce anterior neuroectoderm, while additional active inhibition of the two TGFβ pathways drives them to the posterior fate. Further, all treatment conditions involving the BMP inhibitor LDN‐193189 blocked RPE maturation in hESCs^WT^ lines, indicating that the additional inhibition of the BMP pathway impedes the normal progression of the RPE differentiation program. In contrast, hESCs^del18q^ lines require additional active inhibition of the pathways to be able to exit the pluripotent state and enter the anterior neuroectoderm lineage. We propose that this is likely due to an endogenously hyperactivated activin/nodal signalling, as already observed in a previous study [18].

The SCENIC regulon analysis provided insight on what may be a driver of the second barrier to differentiation: the lack of activation of the *MITF* regulon in hESCs^del18q^. *MITF* was shown to be not only a specific marker of the RPE cell population but also to play a crucial functional role as an essential modulator of pigmentation and maturation [28, 29]. In line with the findings that Wnt inhibition did enhance the ability of hESCs^del18q^ to generate pigmented RPE, *MITF* has been shown to be regulated by β‐catenin and Wnt is known to play a key role in RPE differentiation [29]. Further research is needed to elucidate the exact nature of the interaction between Wnt signalling and *MITF* in the mutant cells.

The common region of loss on chromosome 18q for the two lines in this study spans 21 Mb, containing approximately 150 coding genes. Amongst these, *ADNP2*, *NFATC1* and *SALL3* are transcription factors that can be linked to developmental processes, making them interesting candidates to explain the phenotype of the mutant cells. *SALL3* plays a critical role in neuronal development and its levels have been reported to predict the differentiation capacity of hiPSC [27, 30, 31]. Notably, hiPSC lines with elevated *SALL3* expression preferentially differentiate into ectoderm, while those with reduced *SALL3* levels predominantly form mesoderm and endoderm [27]. Prior research by our group found that hESCs with 18q deletion have an impaired response to directed neuroectodermal differentiation by dual SMAD inhibition, which in this work we show extends also to spontaneous differentiation. Downregulation of *SALL3* by short hairpin RNA in genetically balanced cells, results in the disruption of gene expression in pathways governing pluripotency and differentiation, including TGFβ and WNT signalling [18], but while *SALL3* expression modulates overall neuroectoderm fate commitment, it does not appear to be responsible for the bias in anteroposterior fate we observe in this work. The regulator of this phenotype remains to be elucidated, but *ADNP2* and *NFATC1* make for interesting targets for further research. *ADNP2 is* a zinc finger protein that is implicated in brain development [32, 33]. Its downregulation results in increased cell death associated with oxidative stress. *NFATC1 is* a member of the NFAT transcription factor family and is known to regulate exit of pluripotency and early lineage specification in the mouse [34]. NFAT signalling is required during the earliest differentiation events in mouse embryogenesis, and its inhibition impairs differentiation into the extraembryonic lineages.

It is also important to bear in mind that both hESCs^del18q^ cell lines harbour duplications of 5q14.2qter and 7p22.3pter alongside the 18q deletion. This is because the loss of 18q, which is variable in size across lines, occurs as a derivative chromosome 18, with the lost region of 18q being replaced by the gains of 5q or 7p^22^. It is therefore possible that genes in the duplicated parts of Chromosomes 5 and 7 are also playing a role in modulating the outcomes of differentiation. For instance, the gain of 5q in VUB13^del18q^ cell line spans the gene *HAND1*, a mesodermal transcription factor essential for heart and mesodermal tissue development. Furthermore, VUB14^del18q^ also acquired a homozygous deleterious TP53 mutation [17], which have been reported to impact the differentiation capacity of the cells [35].

Finally, we observed that the differentiated population of hESCs^del18q^ retained a high percentage of proliferating cells, and in one out of two lines, we detected also residual pluripotent cells. The presence of these cells would pose an important risk in differentiated products used in transplantation. It is remarkable that a subpopulation of the cells was able to remain in the undifferentiated state 60 days after withdrawal of the factors that are key for pluripotency maintenance and indicates that at least a subgroup of these cells has become growth‐factor independent.

There is an increasing body of research focusing on understanding the impact of recurrent genetic abnormalities on the characteristics and function of hPSCs. These abnormalities can undermine their quality and viability for future applications by altering their growth patterns and differentiation potential. For instance, gain of 20q11.21 has been extensively studied, with substantial evidence indicating that hPSCs harbouring this alteration altered the distribution of lineages differentiation in embryoid bodies and hinder the commitment to the neuroectodermal lineage [36, 37]. A recent study demonstrated that hPSCs with an isochromosome 20q (iso20q) are prone to apoptosis and are unable to differentiate into the primitive germ layers during spontaneous RPE differentiation [38]. In this study we provide novel mechanistic insight into the impact of aneuploidy on hESCs differentiation in a clinically relevant cell type and show that differential activity of TGFβ is at the basis of the anterior/posterior neuroectoderm specification in hESCs. Our work contributes to the mapping of the risks associated to genetic abnormalities in hPSC, and highlights the importance of thorough genetic testing of cells prior to their use in research and in a clinical setting.

## Author Contributions

Y.L. carried out all the experiments and bioinformatics analysis unless stated otherwise and co‐wrote the manuscript. M.C.D. and A.H assisted with the mRNA extraction and qPCR. D.A.D, C.J., N.K. and M.C.D. assisted with cell culture and immunostaining, E.C.D.D. assisted with the bioinformatics analysis. M.R. assisted in microscopy and making of the figures. K.S. proofread the paper. C.S. co‐wrote the manuscript and designed and supervised the experimental work.

## Ethics Statement

For all parts of this study, the design and conduct complied with all relevant regulations regarding the use of human materials, and all were approved by the local ethical committee of the University Hospital UZ Brussel and the Vrije Universiteit Brussel (File number: B.U.N. 1,432,020,000,284). All patients donating embryos to derive human embryonic stem cell lines gave written consent.

## Conflicts of Interest

The authors declare no conflicts of interest.

## Resource and Materials Availability

Further information and requests for resources should be directed to the corresponding author, Claudia Spits (claudia.spits@vub.be).

All VUB stem cell lines in this study, including the genetically abnormal sub‐lines and genetically modified lines are available upon request and after signing a material transfer agreement.

## Supporting information


**Table S5.** List of all enriched pathways identified by GSEA comparing RPC to undifferentiated hESCs.

**Table S6.** List of all enriched pathways identified by GSEA comparing RPE to undifferentiated hESCs.

**Table S7.** List of all enriched pathways identified by GSEA comparing RPE to RPC.

**Table S8.** List of all enriched pathways identified by GSEA comparing hESCs^del18q^ RPE to hESCs^WT^ RPE.

**Data S1.** Supporting Information.

## Data Availability

Raw sequencing data of human samples is considered personal data by the General Data Protection Regulation of the European Union (Regulation (EU) 2016/679), because SNPs can be extracted from the reads, and cannot be publicly shared. The data can be obtained from the corresponding author upon reasonable request and after signing a Data Use Agreement. scRNA‐seq data supporting the figures in this paper can be found at the Open Science Framework, as well as all the data supporting all figures in this paper (https://osf.io/cnvgt/).
